# Feasibility of an Ultrasound-Based Method for Measuring Talar Displacement during the Anterior Drawer Stress Test Using a Telos Device: A Preliminary Study

**DOI:** 10.3390/ijerph19042367

**Published:** 2022-02-18

**Authors:** Kaori Tsutsumi, Utayo Nakaya, Yuta Koshino, Mari Tateno, Kazuhisa Matsumoto, Mai Tanaka, Mika Yokoyama, Tatsunori Horie, Mina Samukawa, Tamotsu Kamishima, Harukazu Tohyama

**Affiliations:** 1 Faculty of Health Sciences, Hokkaido University, Sapporo 060-0812, Japan; y-t-1-6@hs.hokudai.ac.jp (Y.K.); mina@hs.hokudai.ac.jp (M.S.); tohyama@med.hokudai.ac.jp (H.T.); 2 Department of Radiological Technology, Hokkaido P.W.F.A.C. Sapporo-Kosei General Hospital, Sapporo 060-0033, Japan; uktiusanz00@gmail.com; 3 Rehabilitation Center, NTT Medical Center Sapporo, Sapporo 060-0061, Japan; 4 Department of Radiological Technology, Nakamura Memorial Hospital, Sapporo 060-8570, Japan; withmtb1024@gmail.com; 5 Department of Radiological Technology, Asahikawa Kosei General Hospital, Asahikawa 078-8211, Japan; kazmat0529@gmail.com; 6 Department of Radiological Technology, Sapporo Medical Center, Sapporo 064-0810, Japan; umekichi0425@gmail.com; 7 Graduate School of Health Sciences, Hokkaido University, Sapporo 060-0812, Japan; mika.yokoyama.616@gmail.com; 8 Department of Radiological Technology, Hokkaido University Hospital, Sapporo 060-8648, Japan; t-horie@huhp.hokudai.ac.jp

**Keywords:** ankle flexibility, ultrasound, ankle sprain, anterior drawer test, talus, tibia

## Abstract

This study was conducted to measured talar displacement using ultrasound during an anterior drawer test (ADT) with a Telos device. Five adults (3 men and 2 women; 8 ankles; mean age: 23.2 y) with a history of ankle sprain and eight adults (5 men and 3 women; 16 ankles; mean age: 22.1 y) without a history of ankle sprain were recruited into a history of ankle sprain (HAS) and a control group, respectively. Talar displacement was observed in response to load forces applied by a Telos device during the ultrasound stress imaging test. The ultrasound probe was placed 5 mm inside from the center of the Achilles tendon on the posterior ankle along the direction of the major axis. The inter-rater reliability for the present method was classified as good and excellent (ICC_(2,2)_ = 0.858 and 0.957 at 120 N and 150 N, respectively) in the control group and excellent (ICC_(2,2)_ = 0.940 and 0.905 at 120 N and 150 N, respectively) in the HAS group, according to specific intraclass correlation coefficient values. We found that talar displacement during the ADT was lower in the HAS group than in the control group. Analysis of the receiver operating characteristic curve revealed that the quantitative ultrasound-based ADT using a Telos device was superior to the X-ray-based test in detecting reduced ankle joint mobility during the ADT (area under the curve of 0.905 and 0.726 at a force of 150 N using ultrasound-based and X-ray-based tests, respectively). Further investigation is needed; nevertheless, this preliminary study suggests that the ultrasound-based quantitative ADT using a Telos device might detect talar displacement more sensitively than the conventional stress X-ray.

## 1. Introduction

Ankle sprains, particularly lateral ligament sprains, are the most common type of sports injury. Up to 70% of patients with a history of severe ankle sprains will eventually develop chronic ankle instability (CAI) [1,2,3]. Joint flexibility, especially in the lower extremities, not only increases performance in both sports and daily living activities, but is also an essential factor in injury prevention [4,5,6,7,8,9]. 

In clinical practice, although the stress X-ray test using a Telos device can quantitatively measure ankle instability [10], a manual anterior drawer test (ADT) is more commonly performed [11,12,13]. The stress X-ray test requires an inconvenient procedure and results in radiation exposure. Lee et al. reported on the use of ultrasound examinations during the manual application of an anterior drawer force to evaluate ankle instability. The results indicated that the anterior talofibular ligament (ATFL) ratio in the stress ultrasound test provides useful information for the diagnosis of chronic ankle instability [13]. However, a manual ADT is difficult to assess quantitatively because it depends not only on the experience and subjective feeling of the examiner, but also on the position of the patient, loading stress, and self-defense reaction [14,15]. Stress ultrasound has the advantage of high sensitivity in the detection of ankle instability and does not involve radiation exposure [13,16]. Additionally, according to a recent report by Liu et al., using an arthrometer during an ADT can sensitively detect ankle flexibility [17]. Therefore, in the present study, we combined the advantages of stress ultrasound and quantitative analysis using a Telos stress device and compared talar displacement during the ADT in participants with and without a history of ankle sprain. We also compared the sensitivity between the ultrasound and X-ray-based stress imaging tests and preliminarily evaluated the feasibility of the present methods as a replacement for the conventional stress X-ray test to detect a difference in anterior talar displacement during the ADT.

## 2. Materials and Methods

### 2.1. Participants

This study was conducted in accordance with the Declaration of Helsinki [18] and approved by the Ethics Committee of the Faculty of Health Sciences, Hokkaido University (approval number: 18–59). Participants received sufficient explanations and provided written informed consent before all experiments were carried out. Eight adults (5 men and 3 women; 16 ankles; mean age: 22.1 y) without a history of ankle sprain were recruited as the control group if they fulfilled the following criteria: (1) no history of fracture, (2) no history of ankle sprain within three years, and (3) no history of repeated ankle sprains. The participants self-evaluated using the Cumberland Ankle Instability Tool (CAIT), which is currently the internationally recommended questionnaire for self-evaluation of subjective ankle instability [2]. The CAIT score questionnaire includes the presence or absence of pain in the ankle, the presence or absence of instability at rest and during various activities, and self-control after rolling over on the ankle [19]. All the participants in the control group all reported CAIT scores of 25 points or higher. Five adults (3 men and 2 women; 8 ankles; mean age: 23.2 y) with a history of ankle sprain were recruited into the history of ankle sprain (HAS) group. All the participants in the HAS group reported CAIT scores of less than 25 points as well as a subjective feeling of pain and instability and a history of giving way or recurring sprains [19]. The exclusion criteria included history of fractures, and sprains within the previous year [2]. All the recruited participants were between the age of 20–30 years to avoid the effect of age difference. The participants were also evaluated using the Karlsson ankle functional score (KAFS) to measure ankle joint function [20]. KAFS can estimate the self-feeling of ankle instability, stiffness, and activities during swelling, running, and work [20]. The estimated sample size was calculated as 8:8 with a power of 0.7 by G*power analysis [21,22].

### 2.2. Procedures

#### 2.2.1. Anterior Drawer Stress Test

Stress was loaded for the talar anterior drawer test using a Telos stress device (GAIII/E; Telos Arztund Krankenhausbedarf GmbH, Hungen, Germany). The front pressure cushion of the Telos device was situated 5 cm above the medial malleolus.

#### 2.2.2. Positioning for the Anterior Drawer Stress Test

**Figure 1:**
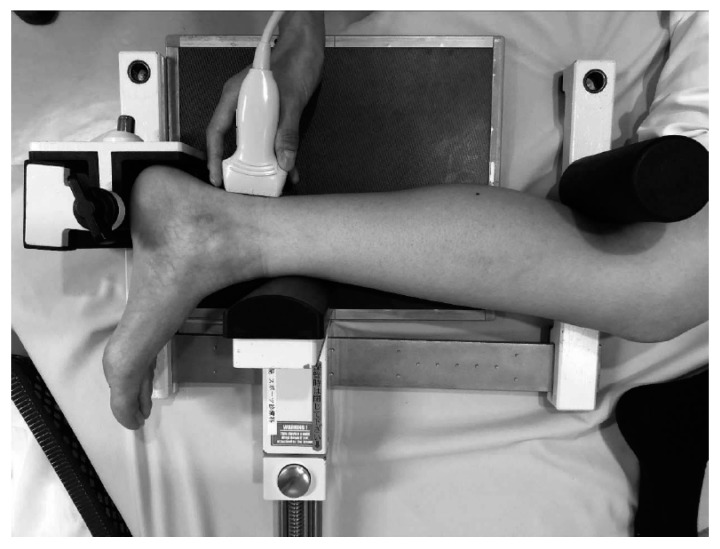
Positioning for the ultrasound stress imaging test and stress X-ray test. The probe was placed 5 mm inside from the center of the Achilles tendon on the posterior ankle along the direction of the major axis. (1) The popliteal fossa of the participant’s posterior knee rested tightly against the pole of the Telos device. (2) The knee joint was bent at 60° with the patella facing anteriorly. (3) The medial malleolus and lateral malleolus were aligned and kept perpendicular to the examination table. (4) The heel was pushed against the Telos device, and the angle of the ankle joint was 0°.

The standard ankle lateral radiograph positioning was used during both the ultrasound and X-ray imaging tests, as follows: the popliteal fossa of the participant’s posterior knee rested tightly against the pole of the Telos device, and the knee joint was bent at 60° with the patella facing anteriorly and the tibia lying parallel to the table; the medial malleolus and lateral malleolus were aligned and kept perpendicular to the examination table; the heel was pushed against the Telos device, and the angle of the ankle joint was 0° (Figure 1).

#### 2.2.3. Ultrasound Stress Imaging Test

**Figure 2:**
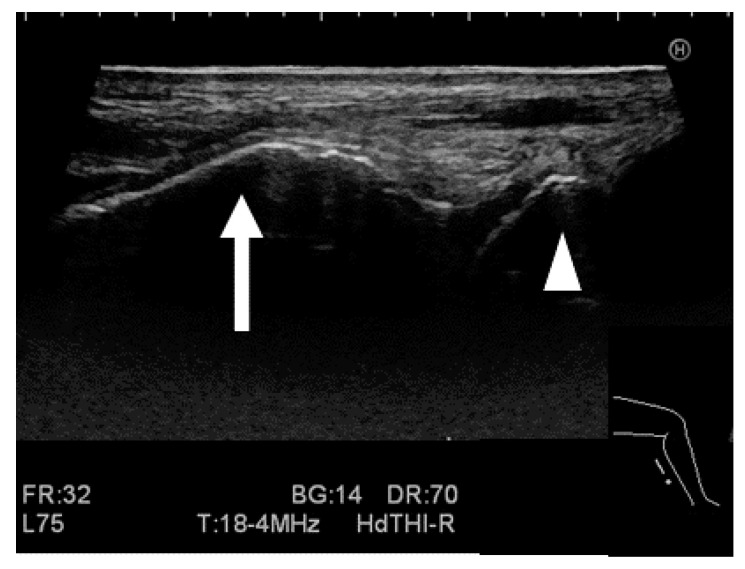
Image of the ultrasound stress imaging test. The arrow represents the edge of the posterior tibial cortical bone, and the arrowhead represents the medial tubercle of the posterior process of the talus.

**Figure 3:**
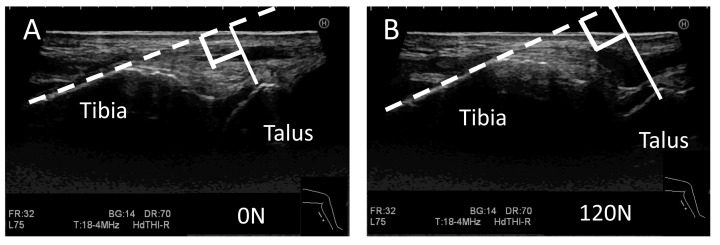
Measurement of the distance between the tibia and talus during the ultrasound stress imaging test. Images from the ultrasound stress imaging test at 0 N (**A**) and 120 N (**B**). The white solid line represents the distance between the tibia and medial tubercle of the posterior process of the talus, and the white broken line represents an extension of the border of the cortical bone of the posterior tibia manually set by the rater.

All ultrasound images were acquired using an Ascendus (FUJIFILM Medical Co., Tokyo, Japan) with an EUP-L75 probe (38 mm linear, 4–18 MHz), and the scanning was focused at a fixed depth of 40 mm. The probe was placed 5 mm inside from the center of the Achilles tendon on the posterior ankle along the direction of the major axis. The examiner adjusted the focus to the posterior process of the talus so that the tibia and the medial tubercle of the posterior process of the talus could be clearly visualized, and the posterior tibia was captured in such a way that it was as long and straight as possible (Figure 1 and Figure 2). The distance between the tibia and talus was measured as the shortest distance between the border of the posterior extension line of the cortical bone on the posterior tibia, which was manually measured by the rater, and the medial tubercle of the posterior process of the talus (Figure 2 and Figure 3). In the control group, ultrasound images were obtained sequentially every 10 N from 0 to 150 N. In the HAS group, ultrasound images were obtained at 0, 120, and 150 N. The anterior displacement of the talus in response to the stress exerted by the Telos device was subsequently measured. The data are expressed as the difference in the tibiotalar distance at the respective stress loads. The distances between the tibia and talus were measured using ImageJ software version 1.48v (National Institutes of Health, Bethesda, MD, USA). 

#### 2.2.4. Stress X-ray Test

**Figure 4:**
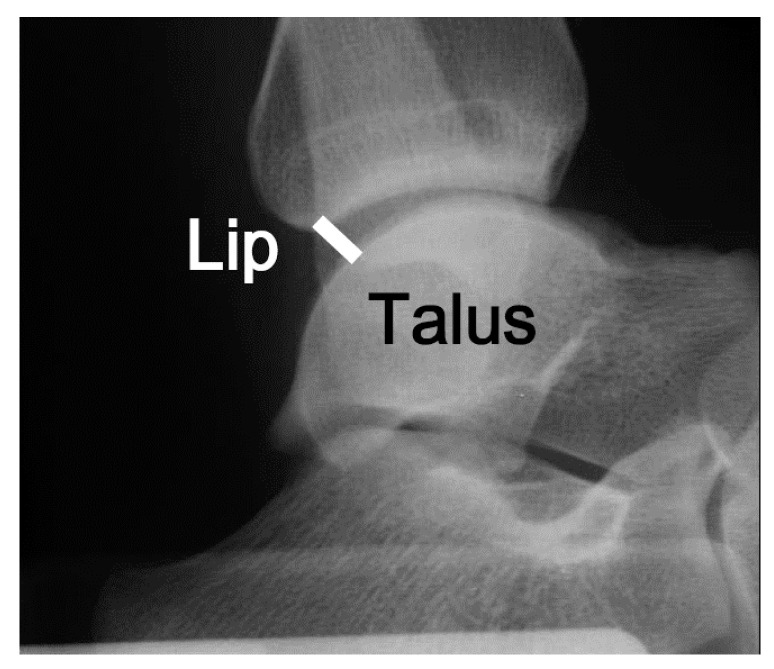
The distance between the tibia and the talus was measured during the X-ray imaging test. The white solid line represents the shortest distance between the tibia and talus. ‘Lip’ indicates the posterior lip of the tibia.

All participants underwent stress radiography. The stress X-ray test was conducted at 0, 120, and 150 N, and images were taken sequentially. We evaluated the distance between the tibia and talus using X-ray images by measuring the shortest distance between the distal portion of the posterior tibia (lip) and the talar border (Figure 4). The amount of anterior talar displacement at 120 and 150 N was calculated with reference to the position at 0 N (Figure 4). Measurements were calculated using RadiAnt DICOM Viewer medical image viewing software version 2020.2.3 (Medixant, Poznan, Poland).

#### 2.2.5. Measurement for the Stress Imaging Test

The examiners who conducted the ultrasound stress imaging test and stress X-ray test were radiologic technologists with more than three years of clinical experience. A separate rater measured the tibiotalar distances on the images taken by the examiners. The examiners physically applied the probes to the participants’ ankle joints and subsequently captured the images. Using these images, the rater determined the shortest distances in millimeters between the tibial cortical bone trailing extensions and the posterior talar process inner nodules. Each examiner acquired the ultrasound and X-ray once on the same day for each participant. This procedure was justified by the inter-rater reliability, as described in the Statistical Analyses. The rater measured the anterior talar displacement three times and was analyzed using the average of them on another day after the stress test without any information about the participants. Neither the examiners nor the raters were informed of the participants’ ultrasound and X-ray image results or self-evaluation scores.

### 2.3. Statistical Analyses

All statistical analyses, including evaluation of normality, calculating a correlation, and a receiver operating characteristic (ROC) curve, were performed using SPSS Statistics version 18 (SPSS Inc., Chicago, IL, USA). Comparisons between the two groups were performed using the Mann–Whitney U test. Differences in the amounts of anterior talar displacement under each load during the ultrasound stress imaging test were analyzed using one-way analysis of variance (ANOVA) with Tukey’s honestly significant difference multiple comparison test. The standard error of the mean was calculated by dividing the standard deviation by the square root of the sample size. Error bars represent standard deviation values and statistical significance was set at *p* < 0.05. Inter-rater reliability between two different raters was calculated based on intraclass correlation coefficients (ICCs) using a two-way random effects model assuming ICC_(2,2)_. The two raters measured the differences in the amounts of anterior talar displacement at 120N and 150N based on 0 N three times and the averages were used to calculate ICCs. The ICCs were classified as follows: values less than 0.50, between 0.50 and 0.75, between 0.75 and 0.90, and greater than 0.90, indicating poor, moderate, good, and excellent reliability, respectively [23]. Correlations between the measurements of anterior talar displacement obtained from ultrasound or X-ray images were estimated using Pearson’s correlation coefficient after verifying normality. R values were classified as follows: values less than 0.20, between 0.20 and 0.40, between 0.40 and 0.70, between 0.70 and 0.90, and greater than 0.90, indicating very weak, weak, moderate, strong, and very strong correlations, respectively [24,25]. 

## 3. Results

### 3.1. Self-Evaluation of Subjective Ankle Instability

**Figure 5:**
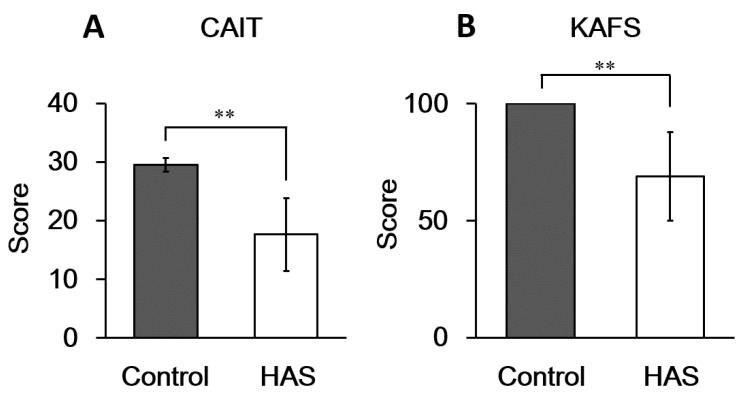
Self-evaluated scores of subjective ankle instability. Average scores reported via (**A**) CAIT and (**B**) KAFS. The HAS group was classified according to self-evaluation without clinical diagnosis. There were 8 subjects in the control group (16 ankles) and 5 subjects in the HAS group (8 ankles). CAIT, Cumberland Ankle Instability Tool; KAFS, Karlsson ankle function score; HAS, history of ankle sprain. ** A statistically significant difference was observed at *p* < 0.01.

First, we compared the scores of self-assessed subjective ankle instability reported in the CAIT questionnaire. The average CAIT score in the control group was 29.5 ± 1.21, and that in the HAS group was 17.7 ± 6.22. The CAIT scores in the HAS group were 60% lower than those in the control group (*p* < 0.01; Figure 5A). The KAFS results were also significantly higher in the control group than in the HAS group (Figure 5B). 

### 3.2. Validation of the Ultrasound-Based Method

**Figure 6:**
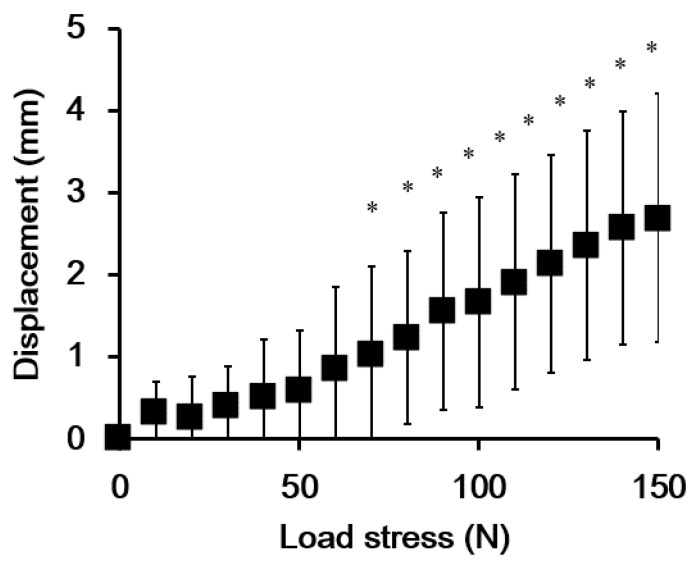
The average amount of anterior talar displacement measured from ultrasound images of participants in the control group (8 subjects, 16 ankles). Error bars represent standard deviation values. * Indicates a significant difference compared to the 0 N baseline condition (*p* < 0.05).

To validate the accuracy of our ultrasound-based method in detecting anterior displacement of the talus during the ADT, we quantified the distances between the talus and tibia under different loads in the control group. The anterior displacement of the talus increased in response to the increased load forces applied by the Telos device (Figure 6). The results of an ANOVA revealed significant anterior displacement at loads > 70 N compared to the 0 N baseline (*p* = 0.012), as shown in Figure 6. 

### 3.3. Inter-Rater Reliability

The inter-rater reliability of the ultrasound stress test was calculated between the two raters. It was classified as good and excellent in the control group, and excellent in the HAS group at loads of 120 and 150 N, respectively (Table 1).

### 3.4. Correlations between Talar Displacement Values Obtained from Ultrasound and X-ray Imaging Tests

We examined the correlations between the amount of anterior displacement of the talus observed by ultrasound imaging and X-ray imaging in the control and HAS groups. Because the measured values showed a normal distribution in both the control and HAS groups, we used Pearson’s correlation coefficient for these comparisons. In the control group, there was a low correlation between the ultrasound stress imaging test and the stress X-ray test at both 120 N and 150 N loads (Table 2). In contrast, in the HAS group, the ultrasound stress imaging and stress X-ray tests showed a high correlation at loads of 120 N (R = 0.814, *p* = 0.026) and 150 N (R = 0.827, *p* = 0.022, Table 2).

### 3.5. Comparison of the Extent of Anterior Displacement of the Talus between the Control and HAS Groups

**Figure 7:**
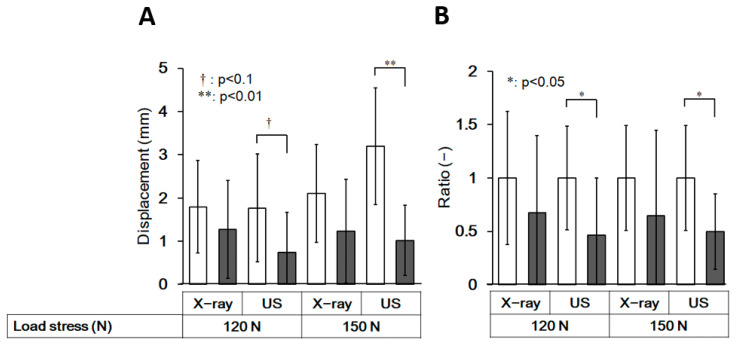
Comparison of the anterior displacement of the talus between the control and HAS groups. The amount of anterior talar displacement (**A**) and the ratio (**B**) compared to the control of each group, measured from stress X-ray and stress ultrasound images (US) in the control group and HAS group at 120 N and 150 N. There were eight subjects in the control group (16 ankles) and five subjects in the HAS group (8 ankles). The open column represents the control group and the closed column represents the HAS group. Error bars represent standard deviation values. The *p*-values were determined using a t-test. HAS, history of ankle sprain; US, ultrasound.

Measurements of anterior displacement of the talus during the ultrasound and X-ray imaging tests were compared between the control and HAS groups (Figure 7). The measurement of anterior displacement of the talus in the HAS group was lower than that in the control group, and it was especially evident at a load of 150 N. Anterior displacement of the talus could be detected with a higher sensitivity by the ultrasound stress test, although there were no significant differences in the conventional stress X-ray test in the HAS group (Figure 7).

### 3.6. Discrimination between Those with and without a History of Ankle Sprain by an Ultrasound Stress Imaging Test

**Figure 8:**
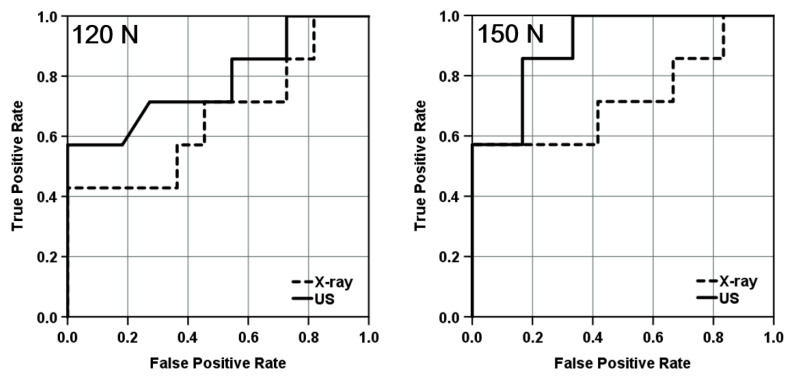
ROC curve indicating sensitivity and specificity of the stress X-ray test and Ultrasound stress imaging test at loads of 120 and 150 N. ROC analysis was performed in the HAS group (*n* = 8). The AUC for the stress X-ray test was 0.662 (*p* = 0.258) at 120 N and 0.786 (*p* = 0.046) at 150 N. The AUC for the ultrasound stress test was 0.786 (*p* = 0.046) at 120 N and 0.905 (*p* = 0.004) at 150 N. ROC, receiver operating characteristic; US, ultrasound; HAS, history of ankle sprain; AUC, area under the curve.

To estimate the sensitivity of the diagnosis of ankle stiffness in the HAS group, we performed ROC analysis and compared the accuracy of diagnosis between the ultrasound stress imaging and stress X-ray tests by calculating the area under the curve, which measures the entire two-dimensional area underneath the ROC curve. Figure 8 shows that the ultrasound stress test at a force of 150 N provided good performance and detection sensitivity for the decrease in the anterior displacement of the talus with a history of ankle sprains.

## 4. Discussion

In the present study, we validated the feasibility of an ultrasound stress imaging test using a Telos stress device to quantitatively detect the anterior displacement of the talus during ADT. This method detected significant talar displacement at stress loads greater than 70 N in the control group (Figure 6). Furthermore, the method detected differences in the anterior displacement measurements between the control and HAS groups with more sensitivity than the conventional stress X-ray test (Figure 7 and Figure 8). The results of this study suggest that ultrasound stress imaging tests using a Telos device might have the potential to detect the anterior talar movement during ADT, more sensitively than stress X-ray tests, with acceptable inter-rater r reliability.

There are several possible reasons why the ultrasound stress imaging test performed better than the stress X-ray imaging test. First, ultrasonography can acquire images while confirming the position and location of the tibia and talus before and during loading stress. However, in stress X-ray imaging tests, it is not possible to confirm the image appropriateness beforehand, and a rescan for a better image is an undesirable option because of increased exposure to X-rays. Second, as X-ray images are summation shadows, it is possible that X-ray imaging tests may not correctly reflect the translocation of the talus by loading stress when the direction of the movement does not coincide with the projection direction of the X-ray image. Third, the talar tends to rotate depending on patient positioning during the ADT using a Telos device [13]; therefore, it can be difficult to identify talar displacement in response to stress from a Telos device. This may be one of the reasons why the correlation between the ultrasound stress imaging test and stress X-ray did not achieve “excellent” values (Table 2). Finally, ultrasonography produces cross-sectional images without overlap of the structures, unlike X-ray imaging, and does not produce a penumbra effect, which leads to blurring in X-rays due to the focal spot.

Lee et al. measured the anterior talofibular ligament (ATFL) length during a manual ADT and compared it with the anterior translation of stress radiography and found that the accuracy of CAI diagnoses using ultrasonography was superior to that of conventional stress X-ray imaging tests [13]. Their findings support our present study regarding the superiority of ultrasound imaging over X-rays for stress testing. In contrast, Lee et al. applied a manual ADT to load stress to avoid talar rotation caused by a Telos device; in the present study, we measured the distance between the tibia and talus as talar displacement by load stress and were able to deduce the influence of talar rotation due to the Telos device. Therefore, we could quantify the load stress controlled by the Telos device, which is a more reproducible method. While the length of the ATFL depends on the shape and thickness of the ligament (e.g., a straight shape versus a bent shape), the tibiotalar distances based on bone–bone distances are very clear and simple. For these reasons, the correlations between stress ultrasound and X-ray imaging tests in the HAS group in the present study were higher than those observed by Lee et al. 

However, in the present ultrasound stress test, two main factors could be the causes of errors, and it is necessary to pay attention to these errors. The first error in the procedure can occur during data acquisition, and the second one can occur during the analytic procedure. The former includes the positioning of the participants, placement of the Telos device, and probe application. Especially to the differences in the angle and the location of the ultrasound probe, it might be necessary to determine the angle of insonation in the long axis to the Achilles tendon or skin surface, not only by visualization of the tibia and the medial tubercle of the posterior process of the talus. In the present study, the ultrasound images were taken by a single examiner and were measured three times by two examiners. The further details of the methods would need to be defined by multiple examiner’s verification in the future. As for the latter, the main source of error could be the drawing way to the extension line of the border of the cortical bone of the posterior tibia, which was set manually by the raters.

We hypothesized that the ultrasound stress imaging test would detect differences in talar displacement during the ADT between participants with and without a history of ankle sprains more sensitively than the stress X-ray imaging test. However, contrary to our expectations, talar displacement in the ADT was significantly decreased in ankles with a history of ankle sprain, especially during the ultrasound stress imaging test, despite the fact that both CAIT scores and KAFS were significantly lower in the HAS group than in the control group (Figure 5 and Figure 7). Ankle sprains generally lead to ankle joint laxity; however, some reports have found that talar hypermobility or no difference from the healthy side, and that patients with acute ankle sprains might exhibit hypomobility [17,26,27]. Furthermore, functional ankle instability, defined mainly by questionnaire assessment, has been shown to not correlate with laxity [27]. Further investigation of talar joint mobility in CAI is needed. 

There are five major limitations to this study that should be addressed in future research. First, we found very weak correlations between the stress X-ray test and the ultrasound stress imaging test in the control group. This may have occurred if ankle flexibility was high and the stretching effect of the ankle was larger in the control group than in the HAS group; that is, stretching in the first stress examination might have affected the subsequent results. To rule out this possibility, the interval between each test should be of sufficient length to prevent interference in the subsequent results. The second limitation of the present study is the small sample size; as a preliminary study, we conducted it with a small sample size, because the ultrasound method we used has not been reported so far, and its detectability is still unknown. However, in reality, it was sufficiently validated with a medium effect size (0.7), as shown in Figure 6. We believe that the difference was more explicit in the ultrasound than in the X-ray test, as demonstrated in Figure 7, which shows the possibility of the usefulness of this method. Larger studies are required to confirm the prevalence of ankle hypomobility after sprain. Third, since our present study adopted only the ADT, further validations with other tests, such as the ankle dorsiflexion range of motion test are needed to verify the ankle flexibility after ankle sprains [17,28]. Fourth, the age range of the participants in the present study was limited to 20 to 30 years. It is necessary to extend the verification to a broader age group in future studies. Finally, because the present study is a preliminary study, it has not been validated in patients clinically diagnosed with ankle instability. To clarify the clinical feasibility of the present ultrasound stress test, it is necessary to verify the results in patients. These findings have the potential to be applied clinically and can be used to quantitatively evaluate small differences in the mechanical mobility of the ankle.

Recurrent sprains are the main cause of CAI, which has deleterious effects not only on sports-related activities, such as declining performance and halting activity, but also on the activities of daily living. To restore normal function, reconstructive surgery and long-term rehabilitation are often required. Therefore, it is necessary to evaluate the differences before the onset of CAI to achieve optimal treatment. The quantification of mobility using the current method may be useful for assessing the risk of developing CAI. Doherty has reported to increase the odds of developing CAI in patients with hypomobility by manual ADT after an acute ankle sprain [26]. In the future, it is necessary to carefully evaluate ankle flexibility after sprain with high sensitivity. Although this method is only in the preliminary experimental stage, the present study could provide basic data on the stiffness of the ankle joint with a history of ankle sprain, and the present ultrasound method has the potential to detect the mechanical differences from the healthy ankle with high sensitivity before CAI without any radiation exposure.

## 5. Conclusions

The results of the present preliminary study suggest that quantitative ADTs performed via ultrasound-based stress imaging tests using a Telos device might detect small differences in the talar anterior displacement more sensitively than the conventional stress X-rays. Although further investigations are essential, the present method has the potential for clinical application to quantitatively evaluate the mechanical mobility of the ankle in the future, conveniently without any radiation exposure.

## Figures and Tables

**Table 1:** Inter-rater reliability of the ultrasound stress test calculated by the ICC_(2,2)_ model ^†^.

	Control				HAS			
Load Stress (N)	ICC_(2,2)_	*p*-Value	95% CI ^‡^	SEM ^§^	ICC_(2,2)_	*p*-Value	95% CI ^‡^	SEM ^§^
120	0.858 **	0.008	0.346–0.968	0.384	0.945 **	0.002	0.702–0.990	0.272
150	0.957 **	0.000	0.857–0.988	0.421	0.905 **	0.007	0.463–0.984	0.298

There were eight subjects in the control group (16 ankles) and five subjects in the HAS group (8 ankles). HAS, history of ankle sprain. ^†^ ICC_(2,2)_ represents the intra-class coefficient using a two-way random-effects model. ^‡^ 95% CI represents the 95% confidence interval. ^§^ SEM represents the standard error of the mean. ** The value is statistically significant at *p* < 0.01.

**Table 2:** Correlation between the anterior talar displacement during stress ultrasound and X-ray tests in the subjects with and without a history of ankle sprain.

	Control		HAS	
Load Stress (N)	R ^†^	*p*-Value	R ^†^	*p*-Value
120	0.306	0.359	0.814 *	0.026
150	0.044	0.898	0.827 *	0.022

There were eight subjects in the control group (16 ankles) and five subjects in the HAS group (8 ankles). HAS, history of ankle sprain. ^†^ R represents Pearson’s correlation coefficient. * The value is statistically significant at *p* < 0.05.

## Data Availability

The data that support the findings of this study are not publicly available. However, data are available from the authors upon reasonable request.
